# Rational Engineering of a Brevinin-2 Peptide: Decoupling Potency from Toxicity Through C-Terminal Truncation and N-Terminal Chiral Substitution

**DOI:** 10.3390/antibiotics14080784

**Published:** 2025-08-01

**Authors:** Aifang Yao, Zeyu Zhang, Zhengmin Song, Yi Yuan, Xiaoling Chen, Chengbang Ma, Tianbao Chen, Chris Shaw, Mei Zhou, Lei Wang

**Affiliations:** 1 College of Biological Science and Engineering, Fuzhou University, Fuzhou 350108, China; 2 Engineering and Technology Institute Groningen, Faculty of Science and Engineering, University of Groningen, Nijenborgh 4, 9747 AG Groningen, The Netherlands; zeyu.zhang@rug.nl; 3 School of Pharmacy, Queen’s University Belfast, 97 Lisburn Road, Belfast BT9 7BL, UK; zsong04@qub.ac.uk (Z.S.); c.ma@qub.ac.uk (C.M.); t.chen@qub.ac.uk (T.C.); chris.shaw@qub.ac.uk (C.S.); m.zhou@qub.ac.uk (M.Z.); l.wang@qub.ac.uk (L.W.)

**Keywords:** antimicrobial peptide, brevinin-2, peptide modification, structure–activity relationship, therapeutic index

## Abstract

**Background/Objectives**: The clinical potential of antimicrobial peptides (AMPs) against dual threats like antimicrobial resistance (AMR) and cancer is often limited by their high host cell toxicity. Here, we focused on brevinin-2OS (B2OS), a novel peptide from the skin of *Odorrana schmackeri* with potent haemolytic activity. The objective was to study the structure–activity relationship and optimise the safety via targeted modifications. **Methods:** A dual-modification strategy involving C-terminal truncation and subsequent N-terminal D-amino acid substitution was employed. The bioactivities and safety profiles of the resulting analogues were evaluated using antimicrobial, haemolysis, and cytotoxicity assays. **Result:** Removal of the rana box in B2OS(1-22)-NH_2_ substantially reduced haemolysis while maintaining bioactivities. Remarkably, the D-leucine substitution in [D-Leu^2^]B2OS(1-22)-NH_2_ displayed a superior HC_50_ value of 118.1 µM, representing a more than ten-fold improvement compared to its parent peptide (HC_50_ of 10.44 µM). This optimised analogue also demonstrated faster bactericidal kinetics and enhanced membrane permeabilisation, leading to a greater than 22-fold improvement in its therapeutic index against Gram-positive bacteria. **Conclusions:** The C-terminal rana box is a primary determinant of toxicity rather than a requirement for activity in the B2OS scaffold. The engineered peptide [D-Leu^2^]B2OS(1-22)-NH_2_ emerges as a promising lead compound, and this dual-modification strategy provides a powerful design principle for developing safer, more effective peptide-based therapeutics.

## 1. Introduction

The dual challenges of rising antimicrobial resistance (AMR) and the search for more effective, less toxic cancer therapies are pressing issues in global public health [1,2]. Conventional treatments have been limited by the rapid evolution of resistance to pathogens and the severe side effects of chemotherapy [3,4]. Antimicrobial peptides (AMPs) are increasingly recognised as promising therapeutic candidates due to their potential to address both threats. Their advantages include broad-spectrum activity, unique mechanisms of action, and a low propensity to induce resistance [5,6]. Despite their therapeutic potential, the narrow therapeutic window is a major problem that limits the clinical development of AMPs [7,8].

The brevinin-2 family, a potent group of AMPs isolated from amphibian skin secretions, exhibits powerful activity against both Gram-positive and Gram-negative bacteria, with some members also displaying wound-healing, anti-cancer and insulin-releasing properties [9,10,11]. Our investigation centres on brevinin-2-OS (B2OS), a novel brevinin-2 peptide isolated and identified from the skin secretion of *Odorrana schmackeri* [12]. Despite its prior discovery, the bioactivities of this peptide are currently uncharacterised. The primary structures of brevinin-2 peptides are poorly conserved, characterised by a disulfide-bridged heptapeptide cyclic motif at the C-terminus named the rana box [13]. The role of the rana box remained ambiguous. The balance of potency and toxicity is known to be highly dependent on the primary sequence and physicochemical properties of the N-terminal domain. For the unique B2OS scaffold, the extent to which the rana box governs its haemolytic activity remained a significant and unaddressed question, presenting a clear opportunity for targeted peptide engineering.

Peptide truncation is a powerful and widely adopted strategy in peptide engineering to refine biological function and enhance the overall therapeutic profile. This strategy can identify minimal functional domains and alter biological activity [14], even yielding shorter analogues with superior efficacy, as function is dictated by the core sequence rather than sheer length [15]. Truncation can also optimise selectivity, producing peptides with reduced toxicity under physiological conditions but enhanced efficacy in specific microenvironments [16]. Given that peptide length is a critical parameter governing biological interactions [17], these precedents provide a strong rationale for using truncation to engineer safer, more potent therapeutics. Concurrently, chiral substitution using D-amino acids represents another key strategy. Incorporating D-amino acid is well established to confer resistance to proteolysis [18], and it also enables the precise modulation of the peptide’s overall physicochemical properties to enhance antimicrobial activity and selectivity [19]. These rational design principles provide a robust toolbox for transforming potent but haemolytic natural peptides into viable clinical candidates.

Guided by these considerations, we devised a two-pronged strategy to design B2OS analogues with a superior therapeutic profile. First, we designed B2OS(1-22)-NH_2_ by excising the C-terminal rana box, attempting to decouple the potent antimicrobial and anti-cancer activities from the undesirable haemolytic effects. Building upon this truncated linear scaffold, our second strategy aims to improve therapeutic selectivity by stereochemistry. We specifically substituted the L-leucine at position 2 with its D-enantiomer, creating [D-Leu^2^]B2OS(1-22)-NH_2_. This site was strategically chosen due to its location within the critical N-terminal region for membrane interaction [20,21] and its low sequence conservation among brevinin-2 peptides [22]. We further hypothesised that it would be a highly tolerant and strategic site for a D-amino acid substitution aimed at modulating membrane selectivity.

Accordingly, this study was designed to synthesise the native B2OS peptide and its two rationally engineered analogues, B2OS(1-22)-NH_2_ and [D-Leu^2^]B2OS(1-22)-NH_2_, for a systematic comparative evaluation. We assessed the in vitro antimicrobial, antibiofilm, and anti-cancer cell proliferative activities, alongside haemolytic activity. Furthermore, kinetic killing and membrane permeabilisation assays were conducted to preliminarily explore their mechanism of action, while the in vivo therapeutic efficacy was assessed using a *Galleria mellonella* infection model. This work aims to clarify the functional roles of the C-terminal rana box and N-terminal chiral substitution in the brevinin-2 scaffold, ultimately delivering a lead compound with a superior balance of potency and safety and advancing the design principles for next-generation peptide-based therapeutics.

## 2. Results

### 2.1. Peptide Conformational Analysis of B2OS and Its Analogues

**Figure 1:**
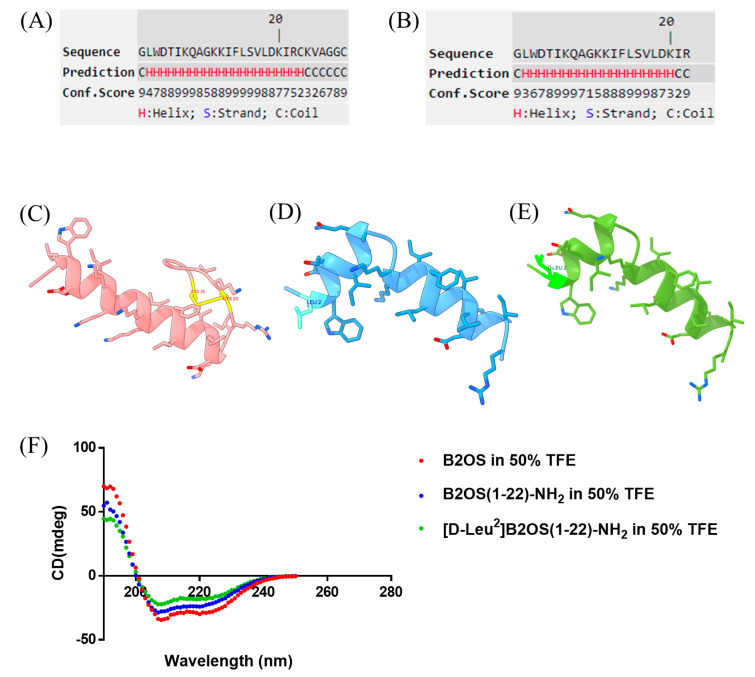
Computational and experimental structural analysis of B2OS and its analogues. Secondary structure prediction of B2OS (**A**) and B2OS(1-22)-NH_2_ (**B**) using I-TASSER. The higher Conf. score means a higher confidence in secondary structure prediction. Color scheme: H (α-helix, red), C (coil, green). S (β-strand, blue) was not predicted in the peptides. The predicted 3D structure models of B2OS (**C**), B2OS(1-22)-NH_2_ (**D**), and [D-Leu^2^]B2OS(1-22)-NH_2_ (**E**) were generated by Pepfold-4 and then visualised using ChimeraX. The CD spectra of B2OS and its analogues in 50% TFE/10 mM NH_4_Ac solution (**F**).

The conformational properties of B2OS and its analogues were first investigated using a computational approach. Initial secondary structure prediction was performed using the I-TASSER server, which suggested that both B2OS and the truncated B2OS(1-22)-NH_2_ were likely to form a predominant α-helical structure (Figure 1A,B). I-TASSER provided high-residue-specific confidence scores for this prediction. The three-dimensional (3D) models were generated by Pepfold-4 and subsequently visualised using ChimeraX (Figure 1C–E). The parameter properties were evaluated by Heli-quest (Table 1). Additionally, the secondary structures of test peptides were further confirmed by the employment of circular dichroism (CD) spectroscopy. As indicated, the parent peptide, B2OS, was characterised by a C-terminal cyclic heptapeptide domain, with a net charge of +4. After removing the rana box, the hydrophobicity was slightly reduced from 0.454 to 0.435, and the net charge was decreased to 3. As shown in Figure 1F, B2OS and its analogues adopted an α-helical structure in 50% TFE, which was consistent with the results of secondary structure prediction.

### 2.2. Minimum Inhibitory Concentration (MIC) and Minimum Bactericidal Concentration (MBC) of B2OS and Its Two Designed Analogues

The antibacterial activities of peptides were evaluated by use of six microorganisms, including *Staphylococcus aureus* (*S. aureus*) (NCTC 10788), MRSA (NCTC 12493), *Enterococcus faecalis* (*E. faecalis*) (NCTC 12697), *Escherichia coli* (*E. coli*) (ATCC 8379), *Klebsiella pneumoniae* (*K. pneumoniae*) (ATCC 43816), and *Pseudomonas aeruginosa* (*P. aeruginosa*) (ATCC 9027). The results of MIC and MBC are summarised in Table 2. The parent peptide, B2OS, could inhibit the growth of *S. aureus* and *E. faecalis* bacteria at a concentration of 8 µM. The MICs of B2OS against Gram-negative bacteria ranged from 16 to 64 µM. As for B2OS(1-22)-NH_2_, a C-terminal cyclic domain deleted peptide, performed relatively well compared to the parent peptide, with MICs ranging from 2 µM to 32 µM. Interestingly, the substitution of L-leucine by D-leucine in the N-terminal of the truncated peptide, [D-Leu^2^]B2OS(1-22)-NH_2_, resulted in a comparable activity to B2OS(1-22)-NH_2_. Collectively, these two engineered analogues exhibited more potent antimicrobial activities against the tested Gram-positive bacteria than against Gram-negative bacteria. This result indicated that the artificial insertion of a D-amino acid at the N-terminal and the removal of the rana box could enhanced the antibacterial activity of brevinin-2 peptides.

### 2.3. Haemolytic Activity of B2OS and Its Analogues

**Figure 2:**
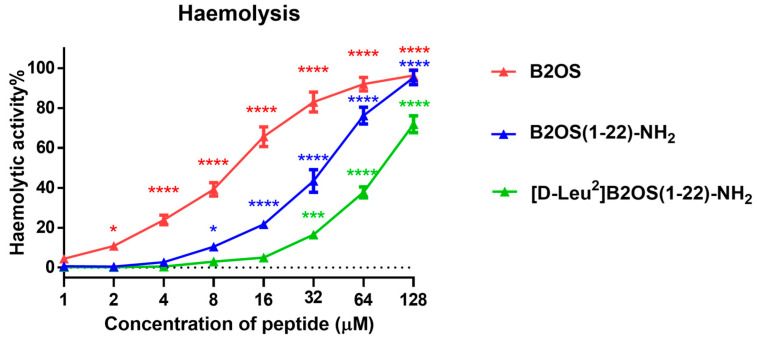
The haemolytic activity of peptides was evaluated by use of horse erythrocytes at a concentration range from 1 μM to 128 μM. Red blood cells treated with 0.1% Triton X-100 and PBS were regarded as the positive and the negative controls, respectively. The statistical significance is indicated as * (*p* < 0.05), *** (*p* < 0.001), and **** (*p* < 0.0001) versus negative control. The error bars are presented as mean ± SEM of three independent experiments.

The haemolytic activity of peptides was evaluated using horse red blood cells, and the 50% haemolytic concentration (HC_50_) and therapeutic index (TI) were summarised in Table 3. As shown in Figure 2, the structural modifications led to a progressive reduction in haemolysis. The parent peptide, B2OS, was highly haemolytic, causing significant lysis at concentrations as low as 4 µM and exhibiting a low TI value around 0.5. In contrast, the truncation of the rana box in B2OS(1-22)-NH_2_ substantially mitigated this toxicity, yielding an HC_50_ value of 41.88 µM. Remarkably, [D-Leu^2^]B2OS(1-22)-NH_2_, which was designed with a D-leucine substitution, was non-haemolytic at concentrations up to 16 µM. This peptide displayed a superior HC_50_ value of 118.1 µM, representing a more than ten-fold improvement compared to its parent peptide (HC_50_ of 10.44 µM). Furthermore, with a TI value of 23.48, [D-Leu^2^]B2OS(1-22)-NH_2_ achieved the highest therapeutic index against Gram-positive bacteria among all synthesised peptides.

### 2.4. Prevention and Eradication of Biofilm by B2OS and Its Two Designed Analogues

In addition to their effects on planktonic cells, the peptides’ ability to inhibit the formation of bacterial biofilms was evaluated. Table 4 summarises the results of the minimum biofilm inhibitory concentration (MBIC) and minimum biofilm eradication concentration (MBEC). The MBICs of the parent peptide, B2OS, against the six microorganisms ranged from 16 μM to 128 μM. The truncated analogues showed notably improved activity against biofilms formed by Gram-positive bacteria. Specifically, B2OS(1-22)-NH_2_ and [D-Leu^2^]B2OS(1-22)-NH_2_ could inhibit the biofilm formation of *S. aureus* and MRSA at 4 μM and 8 μM, which were 8-fold stronger than that of B2OS. Moreover, the modification of D-leucine substitution also improved antibiofilm activity against *E. faecalis* and *P. aeruginosa* 4-fold. These results indicating that the removal of the rana box and the replacement of L-leucine by D-leucine enhanced the antibiofilm potential.

### 2.5. Kinetic Killing Effect of B2OS and Its Analogues

**Figure 3:**
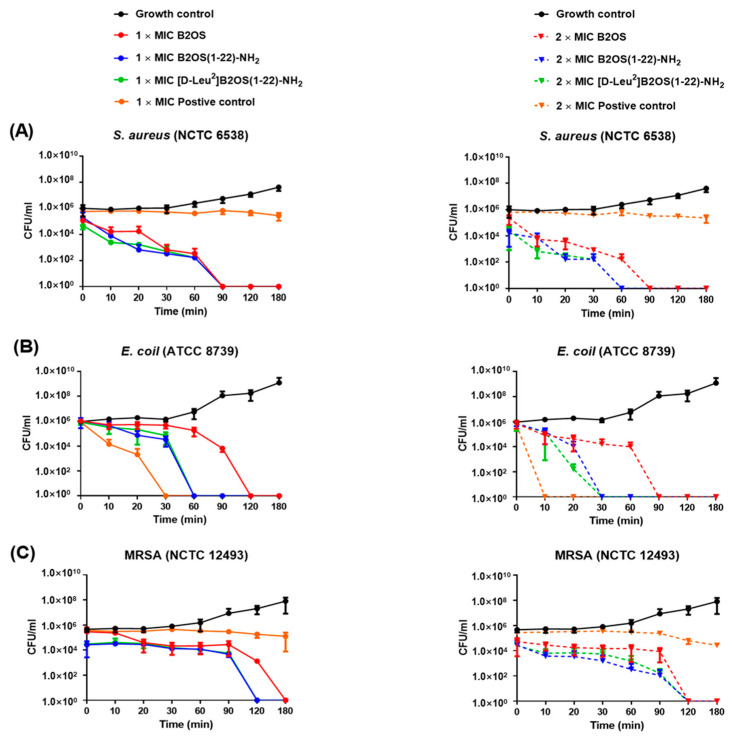
The kinetic time-killing curves of B2OS (red), B2OS(1-22)-NH_2_ (blue) and [D-Leu^2^]B2OS(1-22)-NH_2_ (green) against *S. aureus* (**A**), *E. coli* (**B**), and MRSA (**C**) at concentrations of 1× MIC and 2× MIC. Vancomycin and colistin were used as the positive control for Gram-positive bacteria and Gram-negative bacteria, respectively. The peptide was added at 0 min and monitored until 180 min.

The time-killing assay was conducted to assess the time span of action of different concentrations of B2OS and its analogues on *S. aureus* (NCTC 10788), *E. coli* (ATCC 8739) and MRSA (NCTC 12493). As shown in Figure 3, both truncated analogues induced a more rapid, concentration-dependent bactericidal effect compared to the parent peptide B2OS. Specifically, 1× MIC of [D-Leu^2^]B2OS(1-22)-NH_2_ produced an initial 4.5 × 10^5^ CFU/mL decline in cell counts of *S. aureus* after 20 min, while B2OS and B2OS(1-22)-NH_2_ killed around 1 × 10^5^ CFU/mL of bacteria over the same time. There was no viable *S. aureus* after 90 min incubation. Pathogen cells treated with 2× MIC of B2OS(1-22)-NH_2_ and [D-Leu^2^]B2OS(1-22)-NH_2_ were completely eradicated within 60 min, whereas it took an extra 30 min for B2OS to achieve the same killing effect. As for Gram-negative bacteria (Figure 3B), at a concentration of 1× MIC, B2OS(1-22)-NH_2_ and [D-Leu^2^]B2OS(1-22)-NH_2_ caused a 5 × 10^5^ CFU/mL reduction in cell number of *E. coli* within 60 min, while B2OS achieved the same level of killing in about 120 min. A rapid and significant decline (nearly 5 × 10^5^ CFU/mL drop) in the population of *E. coli* was observed after 20 min treatment by 2× MIC of [D-Leu^2^]B2OS(1-22)-NH_2_. The bacteria could not be counted after 30 min following B2OS(1-22)-NH_2_ and [D-Leu^2^]B2OS(1-22)-NH_2_ exposure, whereas 2× MIC of B2OS eradicated all bacteria after 90 min. As for the drug-resistant bacterium, MRSA, (Figure 3C), the number of colonies forming units was reduced by 2.5 × 10^4^ in the first 90 min at 1× MIC. The truncated peptides, B2OS(1-22)-NH_2_ and [D-Leu^2^]B2OS(1-22)-NH_2_, removed all bacteria within the next 30 min, while the pathogen cell count was nil after 180 min treatment with B2OS. At the concentration of 2× MIC, three of the tested peptides achieved complete eradication of bacteria within 120 min.

### 2.6. Membrane Permeabilisation on MRSA

**Figure 4:**
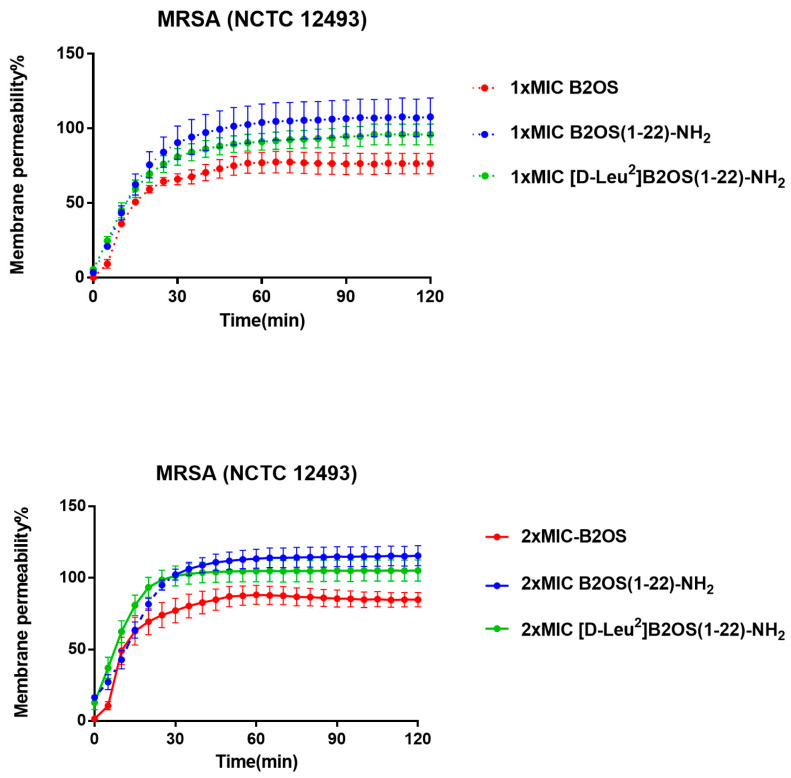
The kinetic membrane permeability curves of B2OS and its analogues against MRSA at concentrations of 1× MIC and 2× MIC. The fluorescence was measured every 5 min. The permeability percentage was obtained by comparing the fluorescent intensity of the bacteria suspension treated with 70% isopropanol. The error bars are presented as mean ± SEM of three independent experiments.

To determine if the rapid bactericidal activity was due to membrane disruption, a key mechanism for many AMPs, we assessed the permeability of peptides on MRSA (NCTC 12493) using the SYTOX Green Nucleic Acid Stain (Figure 4). The results showed that the modified analogues, B2OS(1-22)-NH_2_ and [D-Leu^2^]B2OS(1-22)-MH_2_, induced faster and more extensive membrane permeabilisation than the parent peptide. At 1× MIC, the membrane of MRSA was almost completely permeabilized within 60 min by the modified peptides. In comparison, B2OS reached only about 50% permeabilisation after 30 min incubation, and this rose to 75% membrane disruption after 120 min. Furthermore, as indicated, the integral cell membranes of MRSA were destroyed rapidly and completely after 30 min exposure to 2× MIC of B2OS(1-22)-NH_2_ and [D-Leu^2^]B2OS(1-22)-NH_2_, whereas 2× MIC of the parent peptide, B2OS, could penetrate 85% of membranes of MRSA.

### 2.7. In Vivo Waxworm Larvae Model

**Figure 5:**
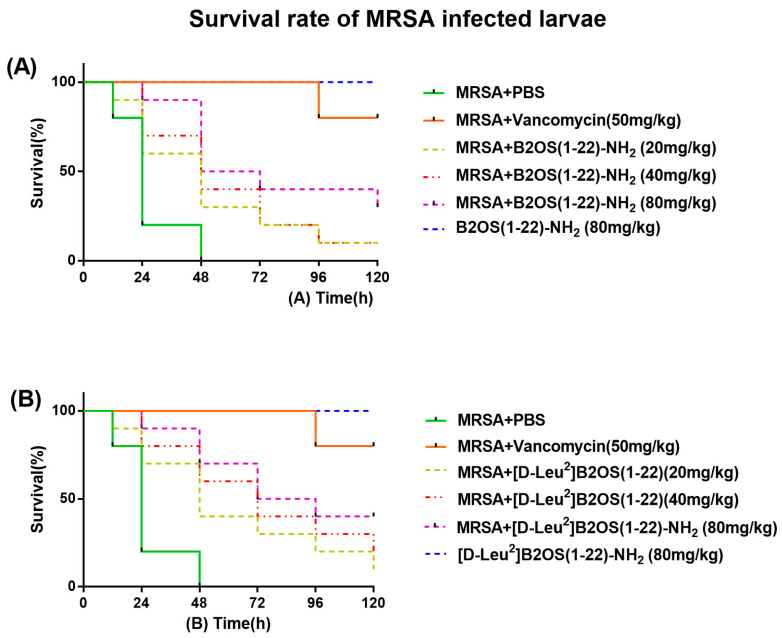
The percentage of survival in waxworm larvae infected with MRSA. The larvae were treated with different doses of (**A**) B2OS(1-22)-NH_2_ and (**B**) [D-Leu^2^]B2OS(1-22)-NH_2_ (20, 40, 80 mg/kg). Sterile phosphate-buffered saline (PBS) and 50 mg/kg of vancomycin were used as negative and positive controls. The larvae with no MRSA were treated with 80 mg/kg of B2OS(1-22)-NH_2_ and [D-Leu^2^]B2OS(1-22)-NH_2_ to evaluate the potential toxicity of peptides to the host.

In the in vitro study, B2OS(1-22)-NH_2_ and [D-Leu^2^]B2OS(1-22)-NH_2_ showed optimised antibacterial activity. Therefore, this attribute was further investigated using the MRSA (NCTC 12489)-infected larvae model. As shown in Figure 5, the mortality of MRSA-infected waxworm larvae was reduced after treatment with peptides. Relatively, [D-Leu^2^]B2OS(1-22)-NH_2_ showed improved in vivo antibacterial activity compared to B2OS(1-22)-NH_2._ A single dose of [D-Leu^2^]B2OS(1-22)-NH_2_ (80 mg/kg) administered post-infection resulted in a significant increase in larvae survival rates over five days compared to the PBS-treated control group, with approximately 30% of larvae surviving after infection. Importantly, administration of the same dose to uninfected larvae showed no signs of toxicity, confirming the favourable safety profile of B2OS(1-22)-NH_2_ and [D-Leu^2^]B2OS(1-22)-NH_2_ at its effective dose in vivo.

### 2.8. Anti-Cancer Proliferation Activity of B2OS and Its Analogues

**Figure 6:**
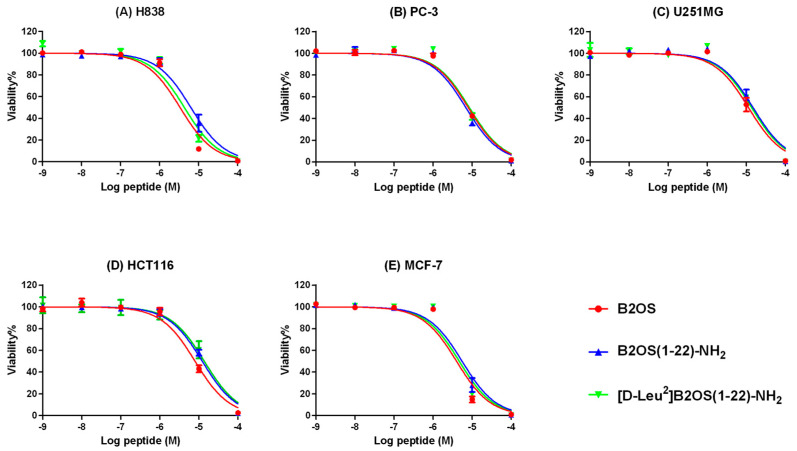
The effect of B2OS (red), B2OS(1-22)-NH_2_ (blue), and [D-Leu^2^]B2OS(1-22)-NH_2_ (green) on the proliferation of human cancer cell lines. (**A**) H838, non-small-cell lung cancer, (**B**) PC-3, human prostate carcinoma cell line, (**C**) U251MG, human glioblastoma astrocytoma, (**D**) HCT116, human colorectal carcinoma cell line, and (**E**) MCF-7, human breast cancer cell line. Cells were treated with peptide for 24 h at a range of concentrations from 10^−9^ to 10^−4^ M. Cells treated with 0.1% Triton X-100 were regarded as the positive controls. The curves were fitted using normalised dose–response analysis. The error bars are presented as mean ± SEM of three independent experiments.

The anti-proliferative activity of B2OS and its analogues was evaluated on human cancer cell lines, including non-small-cell lung cancer cells (H838), human prostate carcinoma cells (PC-3), human neuronal glioblastoma cells (U251MG), human breast cancer cells (MCF-7), and colorectal carcinoma cells (HCT116), in a range of concentrations from 10^−9^ M to 10^−4^ M (Figure 6). In summary, the parent peptide, B2OS, presented broad-spectrum anti-cancer cell activity with half-maximal inhibitory concentration (IC_50_) values between 3.362 µM and 11.52 µM (Table 5). The anti-proliferative activity of B2OS was most pronounced on H838 lung cancer cells. Surprisingly, the C-terminal-truncated peptides maintained this activity but were less potent than the parent peptide. Moreover, [D-Leu^2^]B2OS(1-22)-NH_2_, which was designed by replacing the L-leucine residue with D-leucine at the second position after truncation, displayed comparable anti-cancer cell proliferative activity to B2OS(1-22)-NH_2_.

## 3. Discussion

The parent peptide of this study, brevinin-2OS (B2OS), was previously identified by our group from the skin secretions of *Odorrana schmackeri*, but its biological activities and therapeutic potential remained uncharacterised [12]. This work therefore represents the first systematic evaluation and rational design of this novel brevinin-2 family member. As with many AMPs, the challenge in this work is to uncouple potent bioactivity from inherent host cell toxicity [23]. Our findings demonstrate that the highly haemolytic B2OS could be transformed to [D-Leu^2^]B2OS(1-22)-NH_2_ by C-terminal truncation and N-terminal D-amino acid substitution, with a superior therapeutic profile, defined by a significant increase in selectivity and antimicrobial efficacy.

A particularly noteworthy finding was the exceptionally high haemolytic activity of the parent peptide, B2OS, against horse erythrocytes. While the brevinin-2 family is generally considered to have modest haemolytic activity [24], direct comparisons can be complicated using different erythrocyte species in assays. More relevantly, several independent studies have shown that other members of the brevinin-2 family possess limited haemolytic activity, causing around 20% lysis or less in horse erythrocytes even at concentrations as high as 128 µM [21,25,26]. This finding stands in stark contrast to the potent haemolytic activity exhibited by our parent peptide, B2OS, thereby highlighting its unusual nature. This inherently high toxicity makes B2OS both a challenging and an ideal candidate for engineering. The role of the C-terminal cyclic heptapeptide domain, the rana box, within the brevinin-2 family is not uniform, with conflicting results reported in the literature. For instance, its removal from brevinin-2GHk enhanced antimicrobial activity [26], whereas the same modification in brevinin-2GUb led to a loss of function [21]. Indeed, our results showed that in the B2OS scaffold, the removal of the rana box resulted in improved antimicrobial activity and haemolysis, leading to an enhanced therapeutic index. These observations strongly suggest that the function of the rana box is not absolute. Instead, the biological effects appear to be tightly controlled by the interaction with the unique primary sequence of the N-terminal helical domain. The enhanced therapeutic index after truncation can be attributed to the synergistic optimisation of hydrophobicity and amphipathicity. We observed that the rana box domain (CKVAGGC) of B2OS contains a relatively high content of hydrophobic residues compared to those of other brevinin-2 peptides [22], which likely drives the overall hydrophobicity beyond the optimal threshold for selective activity [27]. We hypothesis that this excessive hydrophobicity is the primary reason for its potent haemolytic activity, as it drives non-specific membrane disruption [28]. Crucially, while the removal of the rana box only slightly decreased the overall hydrophobicity, it enhanced the amphipathicity. This is evidenced by the significant increase in the hydrophobic moment (μH), a key physical parameter that quantifies α-helical amphipathicity, defines the segregation of hydrophobic and hydrophilic face, and modulates the balance between antimicrobial and haemolytic activity [29]. Therefore, we propose that the truncation brought the peptide’s overall hydrophobicity back within the optimal window. This weakens the hydrophobic driving force for non-specific insertion into electrically neutral membranes like those of erythrocytes, resulting in lower haemolytic activity [30]. Concurrently, against this optimised hydrophobic background, the substantially increased hydrophobic moment, representing a more ideal amphipathic structure, could more effectively direct interactions with negatively charged bacterial membranes, thus boosting antibacterial potency.

Beyond improving the therapeutic index, our study revealed that the structural modifications also significantly enhanced the bactericidal kinetics. The time-kill assays showed that the truncated analogues, B2OS(1-22)-NH_2_ and [D-Leu^2^]B2OS(1-22)-NH_2_, eradicate bacteria much more rapidly than the parent peptide. This accelerated killing corresponds directly with their ability to permeabilize the bacterial membrane more quickly and extensively, as demonstrated in the SYTOX Green permeability assay. This finding is supported by studies of other lytic peptides, where truncation has been shown to enhance membrane-disruptive activity under specific environmental conditions, suggesting that removing certain domains can refine permeabilisation [16]. This membrane-targeted mode of action makes peptides less susceptible to drug resistance. It was speculated that the presence of the rana box facilitated increasing membrane affinity rather than participating in transmembrane pore structures [31]. These findings lead us to hypothesise that the N-terminal helix domain of B2OS may be primarily responsible for membrane permeabilisation. According to a previous study, AMPs with a cyclic heptapeptide domain tended to bind tightly to the bacterial outer membrane [26]. The strong membrane affinity restricted further translocation of AMPs on the plasma membrane, resulting in insufficient membrane permeabilisation and a lower killing effect. In contrast, the greater flexibility of the linear truncated peptides may facilitate more efficient translocation, resulting in the observed faster killing. This concept is corroborated by precedents in peptide engineering, where strategically truncated analogues display enhanced efficacy, such as stronger self-assembly, due to modified physicochemical properties like hydrophobicity and conformational flexibility [15]. This enhanced efficiency may also explain their improved antibiofilm activity, as a linear peptide could more effectively penetrate the complex extracellular polymeric substance.

A key finding of our study is the pronounced selectivity of the peptides for Gram-positive bacteria over Gram-negative strains. This differential efficacy is a well-documented phenomenon for many cationic AMPs and is directly linked to the distinct cell envelope architectures of these two bacterial classes [32]. Gram-positive bacteria, such as *S. aureus*, possess a thick cell wall rich in anionic teichoic acids, which creates a strong negative surface charge that electrostatically attracts cationic peptides, facilitating their accumulation and subsequent action [33]. In contrast, the outer membrane of Gram-negative bacteria presents a formidable permeability barrier, primarily due to its outer leaflet being composed almost exclusively of lipopolysaccharide (LPS) [34]. This dense, highly anionic LPS layer can trap peptides, and its disruption is a prerequisite for reaching the inner [35,36]. This difficulty in traversing the protective LPS outer membrane therefore explains the observed preferential activity of our peptides against Gram-positive pathogens.

The most significant result was the further enhancement of selectivity by the N-terminal D-leucine substitution in [D-Leu^2^]B2OS(1-22)-NH_2_. This improvement may be attributed to the stereospecific interaction between the chiral structure and cell membrane components. Evidence has suggested that incorporating D-amino acids is a feasible strategy to optimise the stability and haemolytic activity of AMPs [37,38]. However, such modification strategies tend to have an unfavourable outcome for the conformation [39]. It is therefore crucial to substitute amino acids in the correct positions. Surprisingly, [D-Leu^2^]B2OS(1-22)-NH_2_ displayed comparable antibacterial activity and killing speed to B2OS(1-22)-NH_2_. In addition, the haemolytic activity of [D-Leu^2^]B2OS(1-22)-NH_2_ was far less than the parent peptide. Researchers pointed out that replacing structurally identical amino acids without changing peptide length had little effect on the hydrophobicity of analogues [40]. We propose that introducing a D-amino acid at this specific site caused subtle local conformational change in the α-helix structure but maintained the hydrophobicity. This altered conformation may be less effective at forming lytic pores in cholesterol-rich mammalian membranes while retaining its ability to disrupt the lipid bilayers of bacteria [41]. Overall, our results are in line with previous studies indicating that the artificial addition of D-amino acids into wild-type AMPs, such as pardaxin, melittin, and esculentin-1a (1-21)NH_2_, resulted in lower toxicity and comparable or even better antibacterial activity [42].

Apart from antibacterial activity, the anti-cancer proliferative activity of B2OS and its two analogues was evaluated. An interesting finding was a modest reduction in anti-cancer efficacy after the modification of B2OS, which was different from the trend of antimicrobial and haemolytic effects. This suggests that the peptide’s mode of action against cancer cells may be distinct from its antibacterial mechanism. The general susceptibility of many cancer cell lines to cationic antimicrobial peptides (AMPs) is attributed to fundamental biophysical properties of the malignant cell membrane [43]. Cancer cell surfaces are characterised by a higher net negative charge due to the overexpression and externalisation of anionic molecules, most notably phosphatidylserine (PS) [44,45] and highly sulphated glycosaminoglycans (GAGs) like heparan sulphate (HS) [46]. This provides a strong electrostatic attraction for cationic AMPs, representing the primary mechanism for their initial targeting and accumulation on tumour cells. Studies on Ranatuerin-2PLx and Brevinin-1RL1 have shown that the removal of the rana box or the chemical reduction in its disulfide bridge leads to decrease in anti-proliferative effects [47,48], which align with our study, underscoring that the structural integrity of this motif is indispensable for potent anti-cancer activity. We hypothesis that the rana box perhaps acts as a specific recognition motif for cancer cells. The targeting is likely to begin with an initial electrostatic attraction between the entire cationic peptide and the anionic cell surface, followed by a more specific and higher-affinity interaction mediated directly by the rana box. This unique domain, which often consists of cationic residues within its rigid and constrained cyclic structure, allow it to bind with high affinity to densely clustered anionic targets overexpressed on the cancer cell surface. Therefore, it is speculated that the intact rana box endows B2OS with this enhanced, higher-affinity binding, making it more potent against cancer cells. In contrast, a truncated analogue lacking this specific recognition motif would rely solely on less specific and lower-affinity membrane perturbations along its main peptide body, providing a clear rationale for its reduced efficacy. However, direct experimental validation is required, and future biophysical investigations are warranted to rigorously test this proposed recognition function.

While this study demonstrates the significantly improved therapeutic profile of [D-Leu^2^]B2OS(1-22)-NH_2_, several limitations need to be further studied in future. Firstly, in this study, we evaluated in vivo antibacterial activity and safety in the *Galleria mellonella* model, a comprehensive study in mammalian systems is an essential step to fully assess its pharmacokinetics, biodistribution, and therapeutic performance. Second, it has been reported that the D-amino acid substitution enhances peptide stability, but this property has not been directly evaluated in the current work. Therefore, future studies can confirm this advantage through the protease stability assay. These will be more helpful in developing [D-Leu^2^]B2OS(1-22)-NH_2_ as a viable clinical candidate. Moreover, we acknowledge that this study did not include molecular dynamics (MD) simulations, but this will be addressed in our future studies.

In summary, this study has successfully transformed a highly haemolytic natural antimicrobial peptide, B2OS, into a promising lead candidate, [D-Leu^2^]B2OS(1-22)-NH_2_, with a significantly enhanced therapeutic profile and killing efficacy though a two-step modification strategy. More importantly, our findings establish several key structure–activity relationship principles for the brevinin-2 scaffold. We experimentally demonstrate that the C-terminal rana box domain acts as a primary contributor to haemolysis, rather than a prerequisite for antimicrobial activity. Furthermore, we validate that on a truncated peptide backbone, a single D-amino acid substitution at a strategic N-terminal position is an effective secondary modification to further optimise selectivity and enhance bactericidal kinetics. Therefore, this work not only delivers a promising drug candidate but, more significantly, proposes and validates a dual-modification design principle of truncation for detoxification, followed by strategic chiral substitution for optimisation. This principle offers guidance for rationally engineering other highly haemolysis natural antimicrobial peptides into safer and more effective therapeutic agents.

## 4. Materials and Methods

### 4.1. Peptide Synthesis and Purification

The peptides were synthesised on a Tribute^TM^ automated solid-phase peptide synthesiser (Protein Technologies, Tucson, AZ, USA). The synthesis employed 2-(1H-benzotriazole-1-yl)-1,1,3,3-tetramethyluronium hexafluorophosphate (HBTU) for amino acid coupling activation on either Rink amide MBHA or Fmoc-Cys(Trt)-Wang resin (100–200 mesh) (Millipore Sigma, Burlington, MA, USA). After synthesis, the peptides were cleaved from the side resin and all sidechain-protecting groups were removed with a 2 h incubation in a solution containing 94% TFA (Trifluoroacetic acid), 2% water, 2% TIS (Triisopropylsilane), and 2% EDT (1,2-Ethanedithiol). The resulting crude peptides were precipitated in cold diethyl, washed three times, and lyophilised.

The crude peptides were purified by RP-HPLC system (Waters^®^, Milford, MA, USA) featuring a Phenomenex Aeris 5 µm Peptide Xb-C18 Column (250 × 21.2 mm). Elution was carried out with a linear gradient of 100% buffer A/0% buffer B to 100% buffer B/0% buffer A at a flow rate of 8 mL/min. The identify of fractions were confirmed with a matrix-assisted laser desorption/ionisation time-of-flight (MALDI-TOF) mass spectrometer (Perspective Biosystems, Framingham, MA, USA) using α-cyano-4-hydroxycinnamic acid (CHCA) matrix (10 mg/mL). The HPLC chromatograms (Figure S1) of the purified peptides and corresponding mass spectra (Figure S2) were provided in the Supplementary Document.

### 4.2. Bioinformatics Analysis

A multi-step bioinformatics approach was employed to analyse the structural and physicochemical properties of B2OS and its analogues. First, to obtain a robust initial secondary structure prediction, the sequences were submitted to the I-TASSER server [49,50,51]. This provided a residue-by-residue prediction (e.g., helix or coil) along with a confidence score for the prediction’s reliability. Subsequently, the 3D models were generated using the Pepfold-4 online server (https://mobyle2.rpbs.univ-paris-diderot.fr/cgi-bin/portal.py#forms::PEP-FOLD4 (accessed on 1 June 2024)). The parameters of 3D model generation were set as follows: Generator: fbt; Number of models: 100; Monte Carlo steps: 30,000; Monte Carlo temperature: 370; Pseudo-random seed: 1. The resulting 3D models were visualised using UCSF ChimeraX [52]. The physicochemical properties of B2OS and the two designed shortened peptides were analysed by submitting the sequences to Heli-quest (available online: https://heliquest.ipmc.cnrs.fr/cgi-bin/ComputParams.py (accessed on 1 June 2024)).

### 4.3. Circular Dichroism Spectra

The secondary structures of the peptides were investigated using a JASCO-815 CD spectrometer (JASCO Inc., Tokyo, Japan). Peptides were dissolved in 50% trifluoroethanol (TFE) (*v*/*v* in 10 mM ammonium acetate (NH_4_Ac)) with the final concentration of 50 μM. Next, the prepared peptide samples were loaded into a 1 mm path-length cuvette, respectively. The solution was analysed at room temperature within the range of 190 to 250 nm at a scanning speed of 200 nm/min, a bandwidth of 1 nm, and a data pitch of 1 nm.

### 4.4. Antibacterial Assay

The MIC and MBC assays were conducted to evaluate the inhibitory and bactericidal activities of B2OS, B2OS(1-22)-NH_2_, and [D-Leu^2^]B2OS(1-22)-NH_2_ using six microorganisms, including the Gram-positive bacteria *S. aureus* (NCTC 10788), MRSA (NCTC 12493), and *E. faecalis* (NCTC 12697), as well as the Gram-negative bacteria *E. coli* (ATCC 8379), *K. pneumoniae* (ATCC 43816), and *P. aeruginosa* (ATCC 9027). Peptide stock solutions were made in PBS (512 × 10^2^ μM) and diluted two-fold to achieve a concentration range from 256 × 10^2^ μM to 100 μM. The bacteria in the logarithmic growth phase were diluted to 5 × 10^5^ CFU/mL and co-incubated with the peptides at 37 °C for 20–24 h. The sterile broth was used as the negative control, while vancomycin and colistin were used as the positive control for Gram-positive bacteria and Gram-negative bacteria, respectively. The absorbance was determined at 550 nm using a Synergy HT plate reader (Bio Tek, Washington, DC, USA), with the MIC defined as the lowest peptide concentration showing no visible growth. To establish the MBC, 10 µL aliquots from any wells that appeared clear were plated onto MHA agar and incubated overnight at 37 °C. The experiment was repeated three times independently.

### 4.5. Haemolysis Assays

The haemolytic potential of B2OS and its derivatives was assessed using a 2% (*v*/*v*) horse blood erythrocyte suspension (TCS Biosciences Ltd., Buckingham, UK). A range of peptide concentrations from 256 µM to 2 µM was prepared through two-fold serial dilutions. For this assay, 100 µL of erythrocyte suspension were combined with an equal volume of each peptide concentration and subsequently incubated for 2 h at 37 °C. Samples containing 0.2% Triton X-100 and PBS served as the positive control and negative controls. After incubation, the mixture was centrifuged (930× *g*, 10 min) and 100 μL of the supernatant was transferred to a 96-well plate. The absorbance was measured on a Synergy HT plate reader. The percentage of haemolysis was calculated using the following formula: haemolysis ratio = [absorbance (experimental groups) − absorbance (negative control))/[absorbance (positive control) − absorbance (negative control)] × 100%.

### 4.6. Biofilm Inhibition Assay

The anti-biofilm capabilities of B2OS and its analogues were evaluated by determining their MBIC and MBEC against the microorganisms. For the MBIC assay, peptide solutions (1 μL, ranging from 512 × 10^2^ μM to 100 μM) were co-incubated with a bacterial suspension (99 μL, 5 × 10^5^ CFU/mL) at 37 °C overnight. For the MBEC assay, mature biofilms were first grown in a 96-well plate on a shaking incubator at 37 °C. After planktonic cells were removed by two washes with sterile PBS, the biofilms were exposed to 100 µL of peptide solutions (ranging from 512 µM to 1 µM) and incubated overnight. The remaining biofilm biomass was quantified using a crystal violet staining method. Briefly, wells were washed two more times with PBS and stained with 100 µL of 0.1% crystal violet (Sigma-Aldrich, Gillingham, UK), and the retained stain was dissolved in 100 μL of 30% of acetic acid (Sigma-Aldrich, Gillingham, UK). The absorbance was measured at 595 nm on a Synergy HT plate reader.

### 4.7. Time-Killing Assays

The time-killing kinetic assay was used to determine the kinetic killing of bacteria by various concentrations of B2OS, B2OS(1-22)-NH_2_, and [D-Leu^2^]B2OS(1-22)-NH_2_. *S. aureus* (NCTC 6538), MRSA (NCTC 12493), and *E. coli* (ATCC 8739) were used in this assay. Briefly, 198 µL of diluted bacterial suspension were treated with 2 µL of 2× MIC, 1× MIC of B2OS, B2OS(1-22)-NH_2_, and [D-Leu^2^]B2OS(1-22)-NH_2_, respectively. The bacteria and peptide mixtures were spotted on the MHA agar plate at different time intervals (0, 10, 20, 30, 60, 90, 120 min). After overnight incubation, the colonies of bacteria were counted. The CFU/mL can be calculated using the following formula: CFU/mL = (Number of colonies × dilution factor)/volume of culture.

### 4.8. Bacterial Membrane Permeability Assays

The effect of B2OS, B2OS(1-22)-NH_2_, and [D-Leu^2^]B2OS(1-22)-NH_2_ on the membrane integrity of MRSA (NCTC 12493) was detected by the application of the SYTOX Green Nucleic Acid Stain (Thermo Fisher Scientific, Waltham, MA, USA). MRSA was grown in TSB to the logarithmic growth phase and then centrifuged (1000× *g*, 10 min, 4 °C). The bacterial cell pellet was washed twice with 5% TSB in 0.85% NaCl solution and diluted to 1 × 10^8^ CFU/mL density by measuring the OD value (0.7) at a wavelength of 590 nm. A total of 40 microlitres of B2OS, B2OS(1-22)-NH_2_, and [D-Leu^2^]B2OS(1-22)-NH_2_ at concentrations of 1× MIC and 2× MIC were added to a black 96-well plate. To each well, 10 μL of SYTOX green-fluorescent nucleic acid stain (50 µM, Life Technologies, Renfrew, UK) and 50 μL of diluted bacterial culture were added. Fluorescence intensity was monitored immediately and kinetically for a 2 h period with a Synergy HT plate reader (excitation at 485 nm, emission at 580 nm). The degree of membrane permeabilisation was expressed as a percentage relative to the fluorescence signals from a 100% permeabilisation control (70% isopropanol) and a growth control (5% TSB in 0.85% NaCl).

### 4.9. Efficacy Evaluation of Peptides Against MRSA in Larvae

The in vivo antibacterial efficacy of B2OS(1-22)-NH_2_ and [D-Leu^2^]B2OS(1-22)-NH_2_ against MRSA was evaluated using a *Galleria mellonella* infection model, based on a prior study [53]. The waxworms (Livefood UK Ltd., Rooks Bridge, UK) weighing 250 ± 50 mg were inoculated with 10 μL of MRSA suspension (1 × 10^7^ CFU/mL). After 1 h infection, each waxworm was administrated a 10 µL dose of B2OS(1-22)-NH_2_ or [D-Leu^2^]B2OS(1-22)-NH_2_ at concentrations of 20, 40, or 80 mg/kg. The negative control group was treated with an equivalate volume of PBS, while a positive control group received 50 mg/kg of vancomycin. Each experimental group contained 10 larvae, and larvae survival was monitored by recording the numbers of survivors every 12 h for five days.

### 4.10. Anti-Cancer Cell Proliferation Assays

An MTT (3-(4,5-Dimethylthiazol-2-yl)-2,5-Diphenyltetrazolium Bromide) assay was employed to assess the anti-proliferative activity of B2OS and its analogues. The human cancer cell lines, non-small-cell lung cancer cell line, H838 (ATCC^®^ CRL-5844™), human prostate carcinoma cell line, PC-3 (ATCC^®^ CRL-1435™), human breast cancer cell line, MCF-7 (ATCC^®^ HTB-22™), and human colorectal carcinoma cell line, HCT116 (ATCC^®^ CRL-247™) were obtained from the American Type Culture Collection (ATCC, Manassas, VA, USA). The human glioblastoma astrocytoma cell line U-251 MG (catalogue no. 09063001) was obtained from the European Collection of Authenticated Cell Cultures (ECACC, Porton Down, Salisbury, UK). The cells were seeded at a density of 8000 cells/well in 96-well plates and allowed to adhere overnight (37 °C, 5% CO_2_). After a 4 h serum starvation, the cells were treated for 24 h with 100 μL of peptide solutions prepared in serum-free medium (concentration from 10^−4^ M to 10^−9^ M). Wells treated with 0.1% Triton-X 100 or serum-free medium alone were used as positive and growth controls, respectively. For viability quantification, the cells were incubated with 10 µL of 5 mg/mL MTT for 2 h at 37 °C. The medium was then aspirated, and the formazan crystals were dissolved in 100 µL of DMSO. After 5–10 min of shaking, the optical density was measured at 570 nm with a Synergy HT plate reader (Biotech, Minneapolis, MN, USA).

## Figures and Tables

**Table 1:** Structural parameters of B2OS and its analogues.

Name	Sequence	Hydrophobicity	Hydrophobic Moment	Net Charge
B2OS	GLWDTIKQAGKKIFLSVLDKIRCKVAGGC	0.454	0.278	+4
B2OS(1-22)-NH_2_	GLWDTIKQAGKKIFLSVLDKIR-NH_2_	0.435	0.462	+3
[D-Leu^2^]B2OS(1-22)-NH_2_	G(D-Leu)WDTIKQAGKKIFLSVLDKIR-NH_2_	0.435	0.462	+3

**Table 2:** MICs/MBCs of B2OS and its analogues against different microorganisms.

Microorganisms	MICs/MBCs				
	B2OS	B2OS(1-22)-NH_2_	[D-Leu^2^] B2OS(1-22)-NH_2_	Vancomycin	Colistin
*S. aureus* (NCTC 6538)	8/16	2/4	2/2	0.5/0.5	N/A *
MRSA (NCTC 12493)	32/64	8/8	8/8	0.5/0.5	NA
*E. faecalis* (NCTC 12697)	8/16	8/16	8/16	0.5/>64	NA
*E. coli* (ATCC 8739)	16/16	16/16	8/8	N/A	0.125/0.25
*K. pneumoniae* (ATCC 43816)	32/64	16/32	16/16	N/A	16/16
*P. aeruginosa* (ATCC 9027)	64/128	32/64	32/32	N/A	1/2

* N/A: Not applicable.

**Table 3:** HC_50_ and TI values of B2OS and its analogues.

Peptides	HC_50_ ± 95% CI (μM) ^1^	TI ^2^		
		TI+	TI−	TI _(all)_
B2OS	10.44 (8.428–12.93)	0.82	0.33	0.52
B2OS(1-22)-NH_2_	41.88 (31.03–56.52)	8.31	2.08	4.15
[D-Leu^2^]B2OS(1-22)-NH_2_	118.1 (48.36–288.1)	23.48	7.381	13.15

^1^ The HC_50_ value refers to the concentration of the peptides that can cause 50% erythrocyte lysis. Data are presented as HC_50_ values with 95% confidence intervals (95% CI) in parentheses, derived from three independent experiments. ^2^ The TI was determined as the ratio of HC_50_ to the geometric mean of the MIC values of the corresponding strain group.

**Table 4:** MBICs/MBECs of B2OS and its analogues against different microorganisms.

Microorganisms	MBICs/MBECs		
	B2OS	B2OS(1-22)-NH_2_	[D-Leu^2^]B2OS(1-22)-NH_2_
*S. aureus* (NCTC 6538)	32/512	4/256	4/128
MRSA (NCTC 12493)	64/>512	8/128	8/128
*E. faecalis* (NCTC 12697)	32/>512	16/>512	8/512
*E. coli* (ATCC 8739)	32/256	16/256	16/128
*K. pneumoniae* (ATCC 43816)	64/512	32/256	32/256
*P. aeruginosa* (ATCC 9027)	128/>512	64/>512	32/512

**Table 5:** The IC_50_ values and their 95% CI of B2OS and its analogues against human cancer cell lines.

Cell Line	IC_50_ ± 95% CI (µM) ^1^		
	B2OS	B2OS(1-22)-NH_2_	[D-Leu^2^]B2OS(1-22)-NH_2_
H838	3.624 (2.756–4.101)	6.363 (5.013–8.078)	4.339 (3.242–5.808)
PC-3	8.065 (6.777–9.598)	7.004 (5.685–8.630)	8.551 (6.932–10.55)
U251MG	11.52 (9.103–14.57)	15.05 (11.90–19.02)	13.88 (9.305–20.69)
MCF-7	4.022 (3.158–5.122)	5.571 (4.238–7.323)	4.719 (3.682–6.049)
HCT116	8.052 (6.622–9.790)	12.29 (10.44–14.47)	13.86 (11.92–16.11)

^1^ Data are presented as IC_50_ values with 95% confidence intervals (95% CI) in parentheses, derived from three independent experiments.

## Data Availability

The original contributions presented in this study are included in the article/Supplementary Material. Further inquiries can be directed to the corresponding authors.

## Supplementary Materials

The following supporting information can be downloaded at: https://www.mdpi.com/article/10.3390/antibiotics14080784/s1. Figure S1: Reverse-phase HPLC chromatogram of purified peptides (A) B2OS, (B) B2OS(1-22)-NH_2_, and (C) [D-Leu2]B2OS(1-22)-NH_2_; Figure S2: MALDI-TOF mass spectra of (A) B2OS, (B) B2OS(1-22)-NH_2_, and (C) [D-Leu2]B2OS(1-22)-NH_2_.
