# Supplementary material for: Rational Engineering of a Brevinin-2 Peptide: Decoupling Potency from Toxicity Through C-Terminal Truncation and N-Terminal Chiral Substitution
Source: Antibiotics (Basel). 2025 Aug 1;14(8):784. doi: 10.3390/antibiotics14080784 (PMC12383088; doi:10.3390/antibiotics14080784)
Supplement: Supplementary file 1 [file antibiotics-14-00784-s001.zip › antibiotics-3754521-supplementary.pdf]

Supplement materials

Rational Engineering of a Brevinin-2 Peptide: Decoupling Potency from Toxicity through C-terminal Truncation and N-Terminal Chiral Substitution

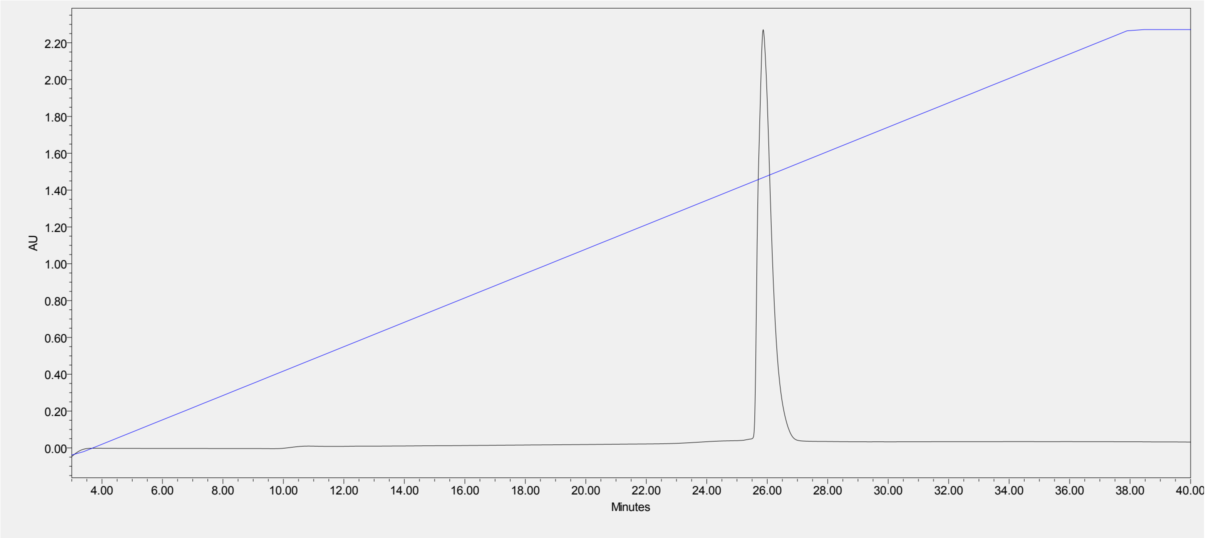

(A)

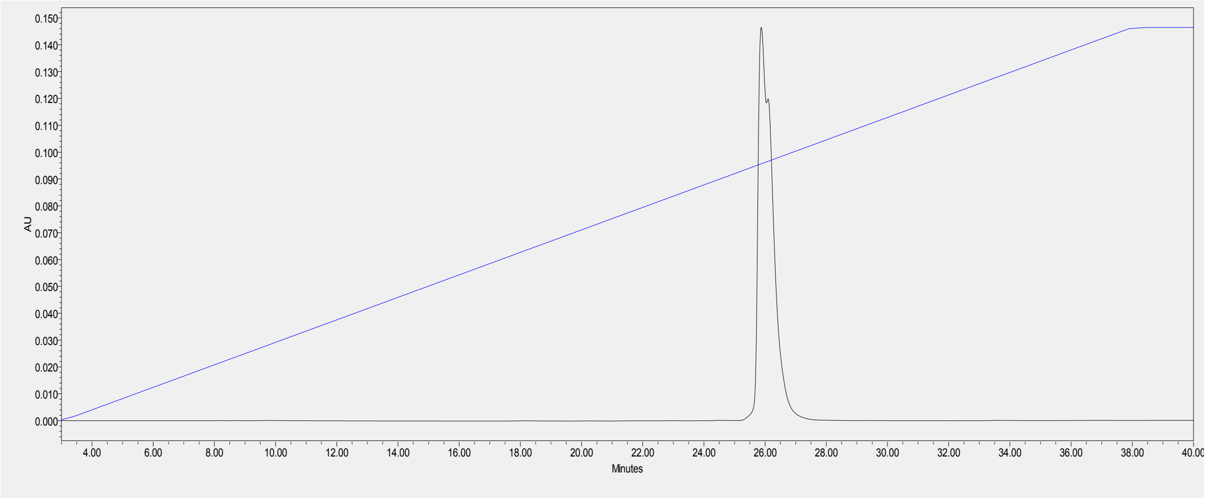

(B)

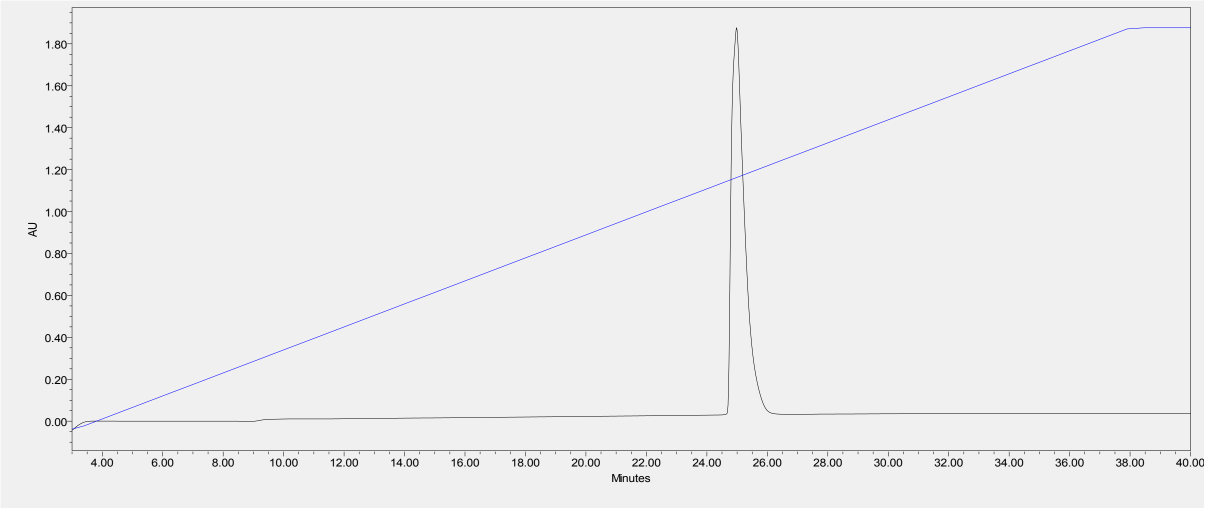

(C)

Figure S1. Reverse phase HPLC chromatogram of purified peptides (A)B2OS, (B)B2OS(1-22)-NH_2, and (C)[D-Leu2]B2OS(1-22)-NH_2 monitored at 214 nm. A liner gradient from 5% to 95% mobile phase B was applied over 35 min (from 3 min to 38 min). Mobile phase A was 0.1% (v/v) trifluoroacetic acid (TFA) in water, and mobile phase B was 0.1% (v/v) TFA in acetonitrile. The peaks indicted the retention time of the pure fragment of the peptide.

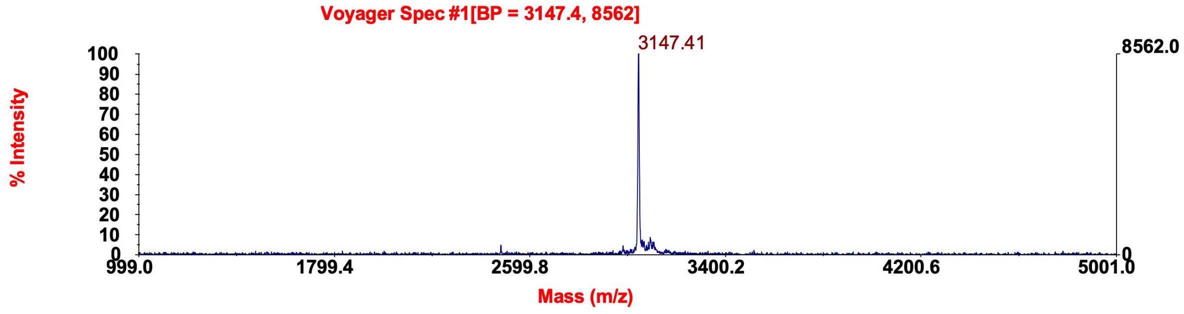

(A)

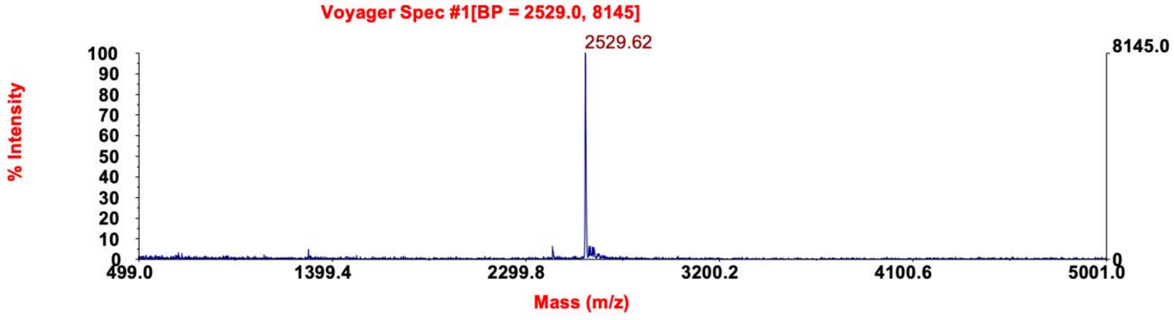

(B)

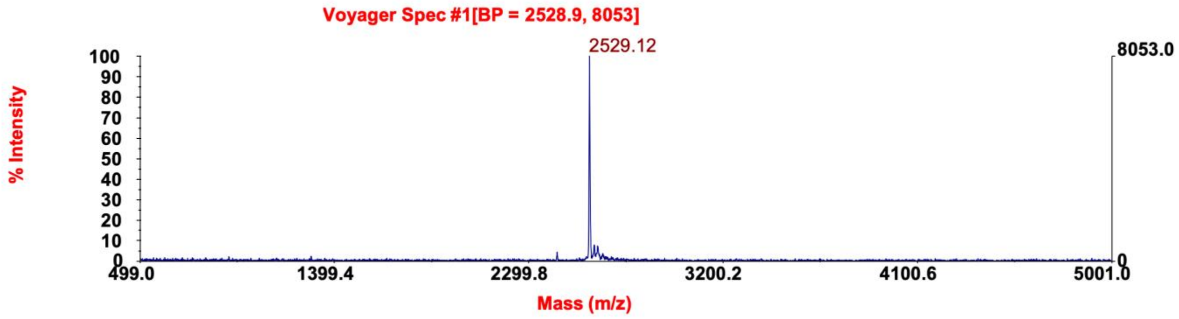

(C)

Figure S2. MALDI-TOF mass spectra of (A)B2OS, (B)B2OS(1-22)-NH_2, and (C)[D-Leu2]B2OS(1-22)-NH_2. The abscissa represents the region of mass-to-charge ratio (m/z). The ordinate indicates the percentage of signal intensity. BP means base peak.
